# Transplant outcomes after intensive chemotherapy or venetoclax plus azacitidine in acute myeloid leukemia: a multicenter retrospective study in Japan

**DOI:** 10.46989/001c.170281

**Published:** 2026-09-22

**Authors:** Hiroyuki Muranushi, Junya Kanda, Tadakazu Kondo, Yasuyuki Arai, Ikue Okamura-Shiki, Takashi Ikeda, Hiroki Amagase, Takehiro Okuda, Daishi Nakagawa, Takeshi Maeda, Yasunori Ueda, Kazunori Imada, Kazuhiro Yago, Tomoharu Takeoka, Mutsumi Okada, Yasuko Miyahara, MItsumasa Watanabe, Nobuyoshi Arima, Toshiyuki Kitano, Masakatsu Hishizawa, Katsuhiro Io, Satoko Oka, Kosuke Asagoe, Takashi Akasaka, Kouhei Yamashita, Akifumi Takaori-Kondo

**Affiliations:** 1 Department of Hematology/Oncology, Kurashiki Central Hospital, Kurashiki, Japan; 2 Department of Hematology, Graduate School of Medicine Kyoto University, Kyoto, Japan; 3 Department of Hematology, Kobe City Medical Center General Hospital, Kobe, Japan; 4 Division of Hematology and Stem Cell Transplantation, Shizuoka Cancer Center, Nagaizumi-cho, Japan; 5 Department of Hematology, Japanese Red Cross Osaka Hospital, Osaka, Japan; 6 Department of Hematology, Shizuoka General Hospital, Shizuoka, Japan; 7 Division of Hematology and Immunology, Japanese Red Cross Otsu Hospital, Otsu, Japan; 8 Department of Hematology, Takatsuki Red Cross Hospital, Takatsuki, Japan; 9 Department of Hematology, Kyoto City Hospital, Kyoto, Japan; 10 Department of Hematology, Hyogo Prefectural Amagasaki General Medical Center, Amagasaki, Japan; 11 Department of Hematology, Shinko Hospital, Kobe, Japan; 12 Department of Hematology, Medical Research Institute Kitano Hospital, Osaka, Japan; 13 Department of Hematology, Kyoto Katsura Hospital, Kyoto, Japan; 14 Department of Hematology, Kansai Electric Power Hospital, Osaka, Japan; 15 Department of Hematology, Japan Red Cross Society Wakayama Medical Center, Wakayama, Japan; 16 Department of Hematology, Shiga General Hospital, Moriyama, Japan; 17 Department of Hematology, Tenri Hospital, Tenri, Japan; 18 Kyoto University

**Keywords:** Acute myeloid leukemia, allogeneic hematopoietic stem cell transplantation, venetoclax, azacitidine

## Abstract

The present retrospective multicenter study in Japan evaluated the role of venetoclax plus azacitidine (V/A) before first allogeneic hematopoietic stem cell transplantation (SCT) in 144 transplant-eligible acute myeloid leukemia (AML). Thirty-seven patients received V/A prior to SCT, mainly as salvage therapy for refractory and adverse-risk AML. Patients were classified into four groups according to remission status at SCT and V/A use. The 1-year overall survival (OS), cumulative incidence of relapse (CIR) and non-relapse mortality (NRM) of those who achieved complete remission (CR) with intensive chemotherapy (IC) were 93%, 5% and 4%, respectively. The 1-year OS, CIR and NRM of those who were refractory/intolerant to IC and achieved CR with V/A were 93%, 7% and 7%, respectively. The 1-year OS, CIR and NRM of those who were refractory to IC and proceeded directly to SCT were 52%, 54% and 13%, respectively. The 1-year OS, CIR and NRM of those who were refractory/intolerant to IC and refractory to V/A were 41%, 39% and 29%, respectively. Overall, use of V/A prior to SCT salvaged some AML patients who were refractory/intolerant to IC, and the transplant outcomes of these patients were favorable. Further studies are needed to optimize treatment for AML prior to SCT.

## 1. Introduction

Acute myeloid leukemia (AML) is an acute onset hematologic malignancy. Intermediate- and adverse-risk AML are often difficult to cure with chemotherapy alone, and allogeneic hematopoietic stem cell transplantation (SCT) is recommended.^1,2^

For many years, the standard treatment for AML was intensive chemotherapy (IC) with anthracycline plus cytarabine.^3^ The high mortality rate due to side effects such as infection and myelosuppression was problematic.^4,5^

Venetoclax plus azacitidine therapy (V/A) has been shown to be effective in the treatment of unfit AML patients,^6^ and became available in Japan in 2020. A retrospective, single-institution analysis from the United States (U.S.) demonstrated that overall survival (OS), cumulative incidence of relapse (CIR) and non-relapse mortality (NRM) were comparable after SCT for AML patients who received V/A versus IC.^7^ There have been similar reports from the U.S. and Europe on the results of pre-transplant V/A,^8–10^ but there have been no reports from Japan. Allogeneic transplants for AML in Japan differ from those in the U.S. and Europe in that cord blood transplants account for approximately 30-40% of allogeneic SCT,^11^ transplants are performed even for non-remission cases,^12,13^ and myeloablative conditioning (MAC) may be selected for patients over 60 years.^14^

In the present study, we performed a multicenter, retrospective analysis of transplant outcomes with pre-transplant V/A for AML and investigated their impact in Japan.

## 2. Methods

### 2.1. Data collection

We retrospectively analyzed the data of 144 AML patients who received 1st allogeneic hematopoietic SCT from January 2021 to February 2023 in the 17 institutes belonging to the Kyoto Stem Cell Transplantation Group. Diagnoses of AML were based on the 5th World Health Organization criteria.^15^ Detailed classifications of AML were based on the 2022 recommendations from an international expert panel on behalf of the European LeukemiaNet (ELN2022).^16^ Diagnoses and assessments of efficacy and toxicity were conducted by the clinicians in each institute. All institutes collaborate with research facilities to conduct diagnoses using next-generation sequencers.^17^

### 2.2. Definitions and Endpoints

Hematological complete remission (CR) was defined as bone marrow blasts < 5%, neutrophil ≥ 1,000/µL and platelets ≥ 100,000/µL. Cytogenetic CR was defined as disappearance of the leukemia-associated cytogenetic abnormality by G-banding or fluorescence *in situ* hybridization. Molecular CR was defined as negativity in polymerase chain reaction for disease-specific transcripts. IC was defined as a multi-day cytarabine-containing regimen at ≥ 100 mg/m^2^ per day, including cytarabine monotherapy,^3^ anthracycline + cytarabine,^3^ anthracycline + cytarabine + etoposide^18^ and anthracycline + cytarabine + fludarabine.^19^ Patients were classified into four groups according to remission status at SCT and V/A use: patients who received IC only and achieved CR before SCT (IC-CR), those who were refractory to IC and proceeded directly to SCT (IC-NonCR), those who were refractory/intolerant to IC and achieved CR with V/A (V/A-CR) and those who were refractory/intolerant to IC and refractory to V/A (V/A-NonCR). Patients who received V/A as induction, salvage, or bridging were included in the V/A-CR or V/A-NonCR group even if they had a history of IC. OS was defined as the duration from transplantation to death, and the patients who remained alive at the final follow-up were censored. The relapse date of patients who did not achieve CR before and after transplantation was defined as day 0. Patients were divided into two groups according to the conditioning regimens: MAC and reduced intensity conditioning (RIC). MAC and RIC were defined according to the previous report.^20^

## 2.3. Statistical analysis

Descriptive statistics were used to summarize variables related to the demographics and clinical characteristics of the patients. Groups were compared using Fisher’s exact test as appropriate for categorical variables and the Kruskal–Wallis test for continuous variables. The Mann–Whitney *U* test was used as a nonparametric test to compare groups. The probabilities of OS and were estimated according to the Kaplan–Meier method, and univariable comparisons among the groups were performed using the log-rank test. The Cox proportional hazard model was used for multivariate analysis of OS. The cumulative incidence rates of relapse and graft-versus-host disease (GVHD) were estimated, and death without these events was considered as a competing factor. NRM was estimated, and relapse was considered as a competing factor. Results were expressed as hazard ratios and their 95% confidence intervals (CI). All tests were two-sided, and a p value of < 0.05 was considered to indicate statistical significance. All statistical analyses were performed using EZR software (Ver. 1.61), which is a graphical user interface for R version 4.2 (The R Foundation for Statistical Computing, Vienna, Austria).^21^

### 2.4. Study Objectives

The primary objective of our study was to clarify the impact of pre-transplant V/A and remission status on one-year OS after transplant in patients who were refractory or intolerant to IC. The secondary objective was to evaluate the impact of pre-transplant V/A and remission status on one-year OS, CIR, NRM and incidence of GVHD in AML patients.

## 3. Results

### 3.1. Patient Characteristics

**Figure 1.**
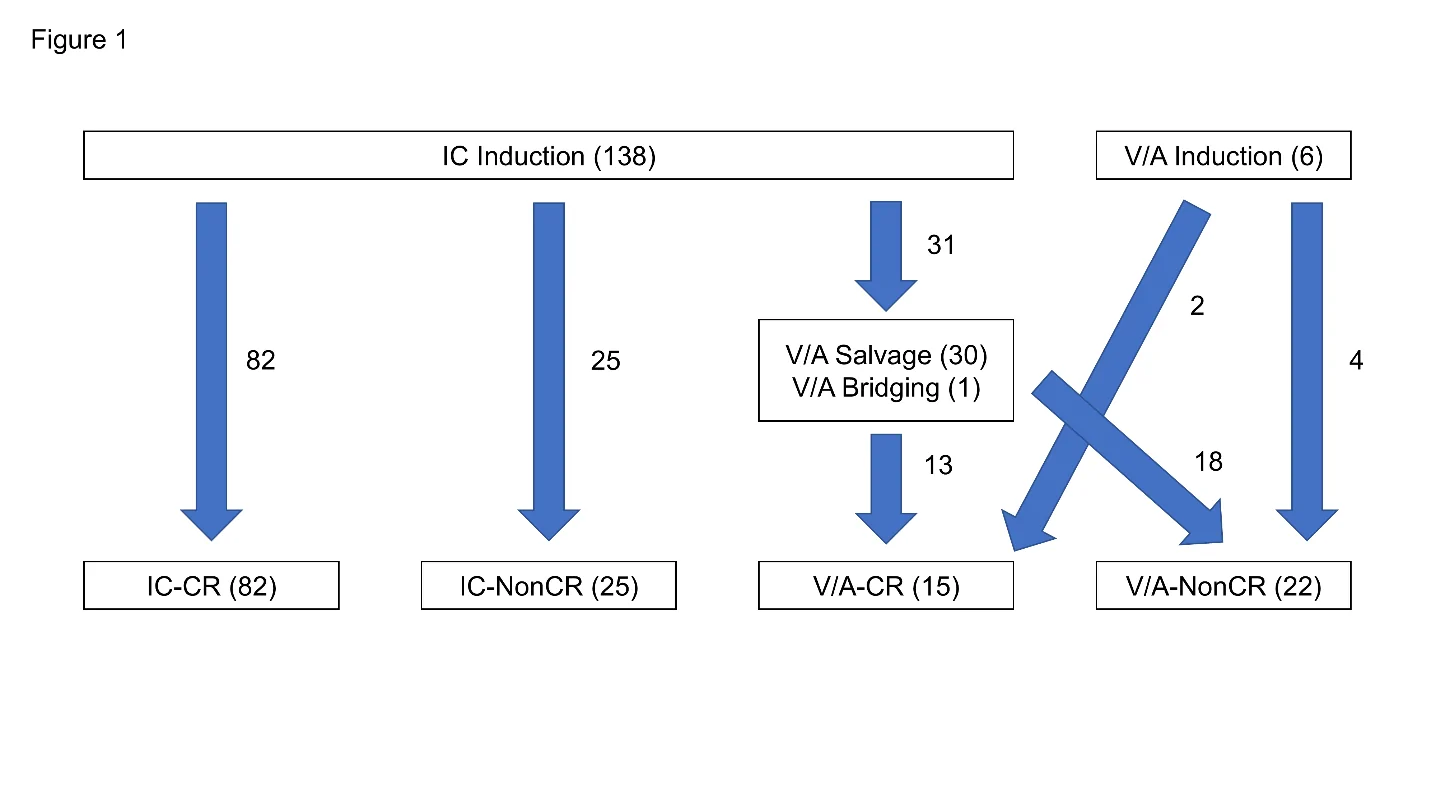
Characteristic of the patients based on their history of intensive chemotherapy and Venetoclax/Azacitidine and their sensitivity to these treatments. IC: intensive chemotherapy, V/A: Venetoclax plus Azacitidine. CR: complete remission.

The characteristics of the patients are shown in Table 1 and Figure 1. In 6 adverse-risk AML patients aged 60 years or older, V/A was administered as induction because the physicians determined that the patients could not tolerate IC. One patient was sensitive to one cycle of IC but unable to continue due to fungal pneumonia and received V/A as bridging. Thirty patients were refractory to IC and received V/A as salvage. The number of IC cycles administered prior to V/A ranged from 1 to 4, with a median of 1. In the V/A-Non-CR group, 16 patients proceeded directly to SCT after V/A, and 6 received 1 or 2 cycles of IC between V/A and SCT. The primary toxicities during pre-transplant V/A were cytopenias, which were all manageable. Non-hematologic toxicities observed during pre-transplant V/A were grade 3 nausea (n =1) and colonic diverticulitis (n =1). Nine patients who received monotherapy with FLT3 (FMS-like receptor tyrosine kinase 3) inhibitors prior to SCT were divided into 4 groups based on their history of IC and V/A and their sensitivity to these treatments. The median age was 55 years old (range: 18-74) and the median observation period for survivors was 1.2 years (range: 0.1-2.6). The most common type of AML was AML not otherwise specified (n = 50). Adverse-risk patients were more common in the V/A treatment groups. Sixty-three patients received cord blood transplantation. Regarding transplants from related donors, only one in the IC-CR group was a bone marrow transplant, while the others were peripheral blood stem cell transplants. One hundred and twelve patients underwent MAC. As for GVHD prophylaxis, 102 patients received tacrolimus plus methotrexate (MTX) or mycophenolate mofetil (MMF), 28 received cyclosporine A plus MTX or MMF, and 14 received post-transplant cyclophosphamide plus tacrolimus and MMF.

**Table 1.** Characteristics of the patients.

		IC-CR n = 82	IC-NonCR n = 25	V/A-CR n = 15	V/A-NonCR n = 22	*p*
Age	Median/Range	53/18-68	59/21-73	55/27-71	60/21-74	0.14
Gender	Male/Female	37/45	10/15	9/6	9/13	0.63
PS	0-1	81 (99)	20 (80)	15 (100)	20 (91)	0.003
	2-4	1 (1)	5 (20)	0 (0)	2 (9)	
Type	NOS	37 (45)	8 (32)	1 (7)	4 (18)	0.16
(ELN2022)	MRCA	10 (12)	9 (36)	5 (33)	6 (27)	
	MRGM	2 (2)	0 (0)	2 (13)	1 (5)	
	*TP53*	0 (0)	1 (4)	4 (27)	3 (14)	
	*RUNX1::RUNX1T1*	8 (10)	1 (4)	0 (0)	0 (0)	
	*CBFB::MYH11*	4 (5)	1 (4)	0 (0)	0 (0)	
	*MLLT3::KMT2A*	2 (2)	0 (0)	1 (7)	1 (5)	
	*GATA2, MECOM*	0 (0)	1 (4)	0 (0)	3 (14)	
	*NPM1*	9 (11)	0 (0)	1 (7)	1 (5)	
	*CEBPA*	2 (2)	0 (0)	1 (7)	2 (9)	
	Others	8 (10)	4 (16)	0 (0)	1 (5)	
Risk	Favorable	18 (22)	2 (8)	2 (13)	2 (9)	0.002
(ELN2022)	Intermediate	43 (52)	13 (52)	3 (20)	5 (23)	
	Adverse	21 (26)	10 (40)	10 (67)	15 (68)	
Cycles of IC Before V/A	0	-	-	2 (13)	4 (18)	0.56
	1	-	-	9 (60)	12 (55)	
	2	-	-	4 (27)	2 (9)	
	3-4	-	-	0 (0)	4 (18)	
Cycles of V/A	1	-	-	3 (20)	11 (50)	0.13
	2	-	-	7 (47)	6 (27)	
	3-4	-	-	5 (33)	5 (23)	
Toxicities of V/A	Grade 3-4 Neutropenia	-	-	15 (100)	21 (95)	1.00
	Grade 3-4 Anemia	-	-	10 (67)	21 (95)	0.03
	Grade 3-4 Thrombocytopenia	-	-	12 (80)	19 (86)	0.67
	Grade 3 Febrile Neutropenia	-	-	3 (20)	6 (27)	0.71
	Grade 3 Nausea	-	-	0 (0)	1 (5)	1.00
	Grade 3 Colonic Diverticulitis	-	-	1 (7)	0 (0)	1.00
Donor	8/8 Related PB/BM	11 (13)	4 (16)	2 (13)	2 (9)	0.60
	7/8 Related PB	2 (2)	0 (0)	0 (0)	0 (0)	
	Haplo Related PB	13 (16)	0 (0)	1 (7)	0 (0)	
	8/8 Unrelated PB	5 (6)	0 (0)	1 (7)	0 (0)	
	7/8 Unrelated PB	1 (1)	0 (0)	0 (0)	0 (0)	
	8/8 Unrelated BM	20 (24)	3 (12)	2 (13)	3 (14)	
	7/8 Unrelated BM	5 (6)	2 (8)	1 (7)	3 (14)	
	Cord blood	25 (30)	16 (64)	8 (53)	14 (64)	
Conditioning	MAC	64 (78)	22 (88)	11 (73)	15 (68)	0.40
	RIC	18 (22)	3 (12)	4 (27)	7 (32)	
GVHD Prophylaxis	Tac-based	57 (70)	22 (88)	10 (67)	19 (86)	0.09
	CyA-based	12 (14)	3 (12)	4 (27)	3 (14)	
	PTCy	13 (16)	0 (0)	1 (7)	0 (0)	

IC: intensive chemotherapy, CR: complete remission, V/A: Venetoclax plus Azacitidine, PS: Performance status, ELN: European LeukemiaNet, NOS: not otherwise specified, MRCA: myelodysplasia-related cytogenetic abnormalities, MRGM: myelodysplasia-related gene mutations, 8/8: 8/8 allele matched, 7/8: 7/8 allele matched, Haplo: Haploidentical, PB: Peripheral Blood, BM: Bone Marrow, MAC: Myeloablative conditioning, RIC: Reduced-intensity conditioning, GVHD: graft-versus-host disease, Tac: Tacrolimus, CyA: Cyclosporine A, PTCy: Post-transplant cyclophosphamide. The numbers in parentheses indicate percentages.

### 3.2. Remission status at transplantation

Among patients who did not receive V/A prior to transplant (n = 107), 23 did not achieve CR, 57 achieved hematological CR, 10 achieved cytogenetic CR, and 15 achieved molecular CR at SCT. Among patients who received V/A prior to transplant (n = 37), 22 did not achieve CR, 12 achieved hematological CR, 2 achieved cytogenetic CR, and one achieved molecular CR at SCT.

### 3.3. Overall survival

**Figure 2.**
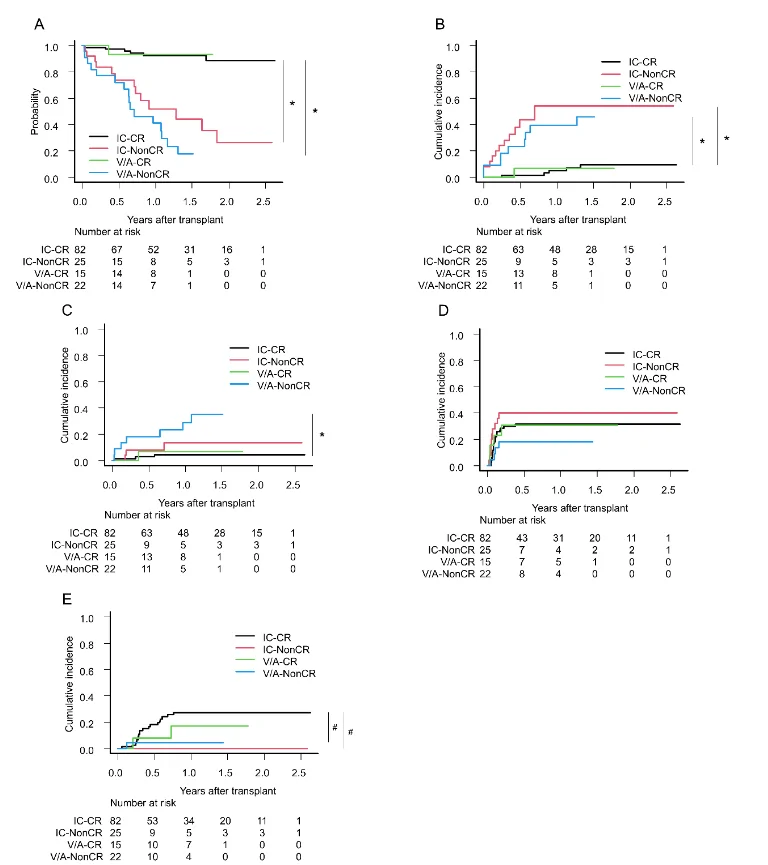
Outcomes of overall patients. A: Overall survival. B: Cumulative incidence of relapse. C: Non-relapse mortality. D: Cumulative incidence of grade II-IV acute graft-versus-host disease. E: Cumulative incidence of chronic graft-versus-host disease. *: p < 0.01, #: p < 0.05. IC: intensive chemotherapy, V/A: Venetoclax plus Azacitidine. CR: complete remission.

The 1-year OS of IC-CR, V/A-CR, IC-NonCR, and V/A-NonCR groups were 93% [95% CI: 83-97%], 93% [95% CI: 61-99%], 52% [95% CI: 28-71%] and 41% [95% CI: 20-61%], respectively (IC-CR versus V/A-CR: p = 0.56, IC-CR versus IC-NonCR: p = 0.001 and IC-CR versus V/A-NonCR: p < 0.001) (Figure 2A).

### 3.4. Relapse, non-relapse mortality, GVHD and maintenance

The 1-year CIR of IC-CR, V/A-CR, IC-NonCR, and V/A-NonCR groups were 5% [95% CI: 1-12%], 7% [95% CI: 0-27%], 54% [95% CI: 30-73%] and 39% [95% CI: 18-60%], respectively (IC-CR versus V/A-CR: p = 0.34, IC-CR versus IC-NonCR: p < 0.001 and IC-CR versus V/A-NonCR: p < 0.001) (Figure 2B).

The 1-year NRM of IC-CR, V/A-CR, IC-NonCR, and V/A-NonCR groups were 4% [95% CI: 1-11%], 7% [95% CI: 0-27%], 13% [95% CI: 3-32%] and 29% [95% CI: 11-50%], respectively (IC-CR versus V/A-CR: p = 0.34, IC-CR versus IC-NonCR: p = 0.20 and IC-CR versus V/A-NonCR: p = 0.005) (Figure 2C).

The data on infection, cardiac failure and non-relapse death after SCT are shown in Table 2. In the V/A-NonCR group, non-relapse deaths were observed in 7 patients, 4 of whom died of infection.

**Table 2.** Infection, cardiac failure and non-relapse death after SCT.

		IC-CR n = 82	IC-NonCR n = 25	V/A-CR n = 15	V/A-NonCR n = 22
Infection	CMV viremia	35 (43)	9 (36)	1 (7)	6 (27)
	COVID-19	3 (4)	0 (0)	0 (0)	1 (5)
	Febrile Neutropenia	63 (77)	18 (72)	9 (60)	19 (86)
	Bacteremia	17 (21)	3 (12)	0 (0)	2 (9)
	Fungal infection	3 (4)	2 (8)	0 (0)	1 (5)
Cardiac failure		2 (2)	1 (4)	0 (0)	1 (5)
	Bacterial Pneumonia	0 (0)	0 (0)	0 (0)	1 (5)
	Fungal Pneumonia	1 (1)	0 (0)	0 (0)	1 (5)
	COVID-19	0 (0)	0 (0)	0 (0)	1 (5)
	Sepsis	0 (0)	1 (4)	0 (0)	1 (5)
	Interstitial Pneumonia	0 (0)	0 (0)	1 (7)	1 (5)
	SOS	0 (0)	1 (4)	0 (0)	0 (0)
	Colon Rapture	1 (1)	0 (0)	0 (0)	0 (0)
	Multiorgan Failure	0 (0)	1 (4)	0 (0)	1 (5)
	Acute GVHD	1 (1)	0 (0)	0 (0)	0 (0)
	Chronic GVHD	0 (0)	0 (0)	0 (0)	1 (5)

IC: intensive chemotherapy, CR: complete remission, V/A: Venetoclax plus Azacitidine, CMV: cytomegalovirus, COVID: Coronavirus Infectious Disease, SOS: Sinusoidal Obstruction Syndrome, GVHD: Graft versus host disease. The numbers in parentheses indicate percentages.

There was no significant difference in the incidence of grade II-IV acute GVHD among the 4 groups (p = 0.38) (Figure 2D).

The 1-year incidence of chronic GVHD of IC-CR, V/A-CR, IC-NonCR, and V/A-NonCR groups were 27% [95% CI: 23-31%], 17% [95% CI: 2-44%], 0% [95% CI: 0-0%] and 5% [95% CI: 0-20%], respectively (IC-CR versus V/A-CR: p = 0.42, IC-CR versus IC-NonCR: p = 0.001 and IC-CR versus V/A-NonCR: p = 0.046) (Figure 2E).

The data on maintenance after SCT are shown in Table 3. Six patients received V/A or venetoclax monotherapy, and one received 2 donor lymphocyte infusions. V/A was effective in 2 patients and venetoclax monotherapy was effective in one.

**Table 3.** Maintenance therapy after transplantation.

Mainte- nance	Age	Sex	AML Type	Risk	Donor	Cond	GVHD Pro	Duration (days)	Final Response
V	26	M	NOS	Int	CB	MAC	TAC	720	CyCR
V	52	F	TP53	Adv	CB	MAC	TAC	28	PD
V	47	M	TP53	Adv	MUBM	RIC	TAC	14	PD
V/A	41	F	TP53	Adv	CB	MAC	CyA	430	hCR
V/A	46	F	GATA2, MECOM	Adv	7/8UBM	MAC	TAC	21	PD
V/A	57	F	TP53	Adv	CB	MAC	TAC	164	CyCR
DLI 2	66	M	MRCA	Adv	MUBM	MAC	TAC	14	PD

AML: acute myeloid leukemia, Cond: Conditioning, GVHD Pro: Graft-versus-Host Disease Prophylaxis, V: Venetoclax, V/A: Venetoclax plus Azacitidine, DLI 2: 2 times of donor lymphocyte infusion, M: male, F: female, NOS: not otherwise specified, MRCA: myelodysplasia-related cytogenetic abnormalities, Int: intermediate, Adv: adverse, CB: cord blood, MUBM: HLA-matched unrelated bone marrow, 7/8UBM: 7/8 allele mismatched unrelated bone marrow, MAC: myeloablative conditioning, RIC: reduced-intensity conditioning, TAC: Tacrolimus-based, CyA: Cyclosporine A-based, CyCR: cytogenetic complete remission, PD: progressive disease, hCR: hematological complete remission

### 3.5. Adverse-risk AML

**Figure 3.**
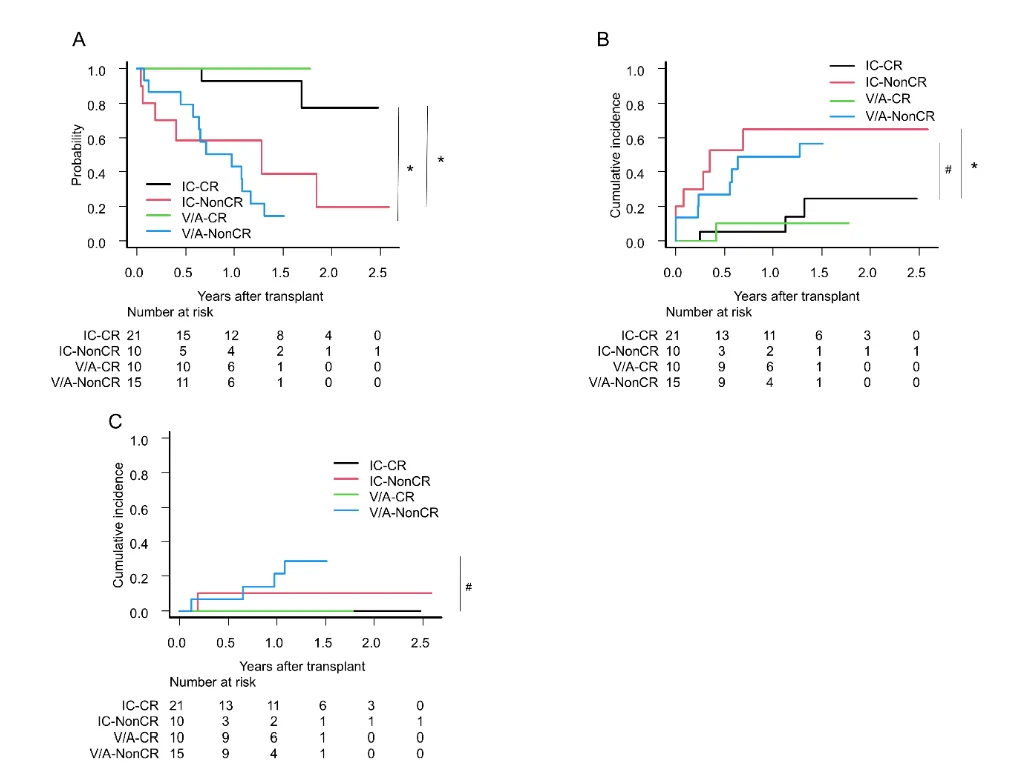
Outcomes of adverse-risk patients. A: Overall survival. B: Cumulative incidence of relapse. C: Non-relapse mortality. *: p < 0.01, #: p < 0.05. IC: intensive chemotherapy, V/A: Venetoclax plus Azacitidine. CR: complete remission.

In the analysis limited to the patients with adverse-risk AML, the 1-year OS of IC-CR, V/A-CR, IC-NonCR, and V/A-NonCR groups were 93% [95% CI: 59-99%], 100% [95% CI: not available due to the absence of events], 58% [95% CI: 23-82%] and 43% [95% CI: 18-67%], respectively (IC-CR versus V/A-CR: p = 0.37, IC-CR versus IC-NonCR: p = 0.003 and IC-CR versus V/A-NonCR: p < 0.001) (Figure 3A).

The 1-year CIR of IC-CR, V/A-CR, IC-NonCR, and V/A-NonCR groups were 5% [95% CI: 0-22%], 10% [95% CI: 0-37%], 65% [95% CI: 21-89%] and 49% [95% CI: 21-72%], respectively (IC-CR versus V/A-CR: unable to compare due to no non-relapse deaths, IC-CR versus IC-NonCR: p = 0.007 and IC-CR versus V/A-NonCR: p = 0.03) (Figure 3B).

The 1-year NRM of IC-CR, V/A-CR, IC-NonCR, and V/A-NonCR groups were 0% [95% CI: 0-0%], 0% [95% CI: 0-0%], 10% [95% CI: 0-38%] and 22% [95% CI: 4-47%], respectively (IC-CR versus V/A-CR: unable to compare due to no non-relapse deaths, IC-CR versus IC-NonCR: p = 0.16 and IC-CR versus V/A-NonCR: p = 0.04) (Figure 3C).

**Figure 4.**
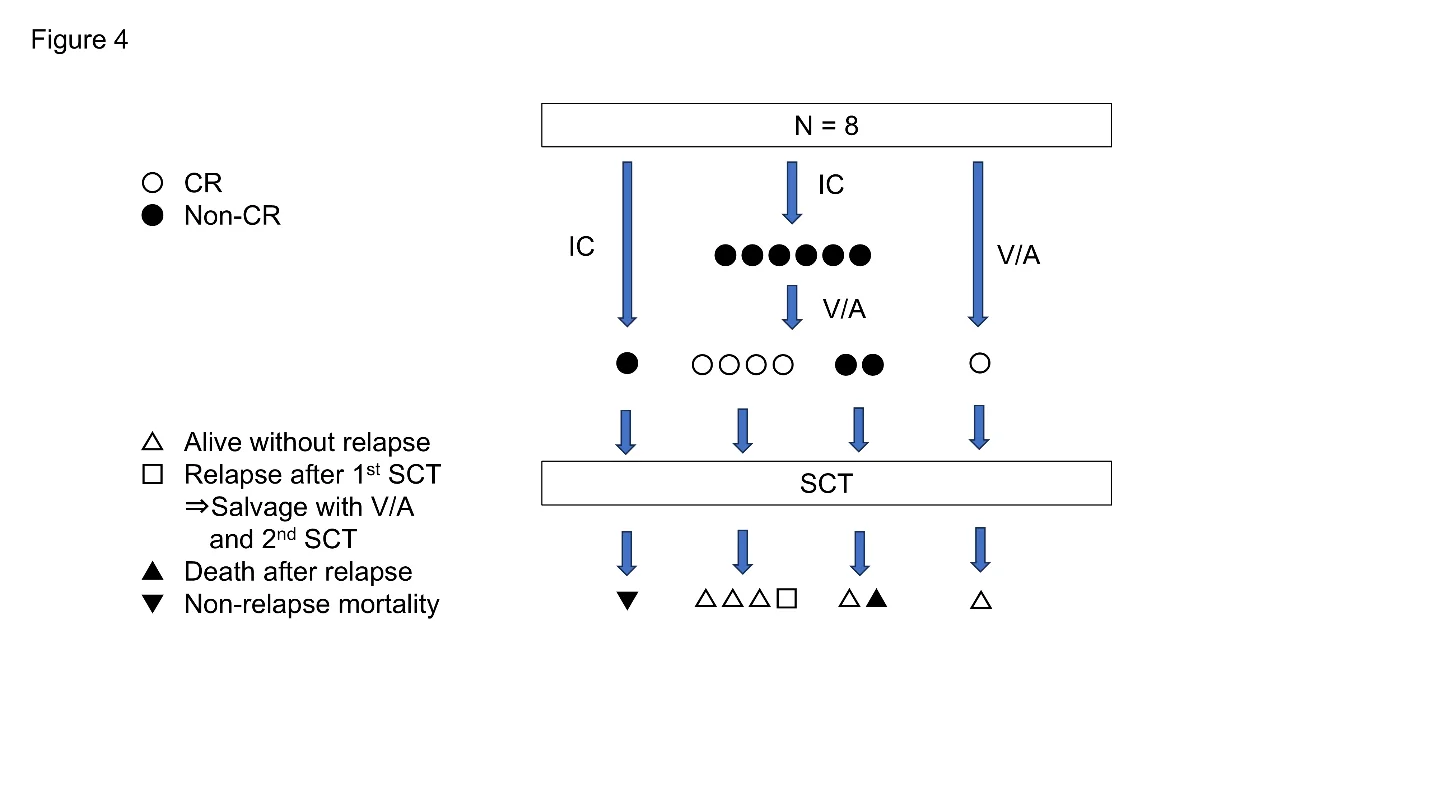
Outcomes of patients with *TP53* mutations. CR: complete remission. SCT: stem cell transplantation. IC: intensive chemotherapy. V/A: Venetoclax plus Azacitidine.

There were 8 patients with *TP53* mutations. IC was ineffective in 7, and one patient proceeded directly to SCT and died of multiorgan failure after SCT. The remaining 6 patients received V/A after IC, and one received V/A as induction. Five patients reached hematological CR after V/A. After SCT, 5 patients survived without relapse. One patient died of relapse after SCT. One patient relapsed after first SCT but achieved hematological CR again with V/A, and a second SCT was performed (Figure 4).

### 3.6. Venetoclax plus azacitidine as induction

Six patients received V/A as induction. Two were in CR prior to SCT; both survived without relapse during the observation period. One patient initially achieved CR but experienced disease progression prior to SCT; the patient subsequently relapsed and died after SCT. Three patients proceeded to SCT without achieving CR. Of these, one survived without relapse during the observation period, while the remaining two died from COVID-19 and fungal pneumonia, respectively.

### 3.7. Multivariate Analyses for overall survival, relapse and non-relapse mortality

NonCR at SCT was the only poor prognostic factor for OS (Table 4). Age < 50, Female to Male SCT, RIC and NonCR at SCT were poor prognostic factors for relapse (Table 5). Age ≥ 50 and NonCR at SCT were poor prognostic factors for NRM. (Table 6).

**Table 4.** Multivariate Analysis for overall survival.

		HR	95% CI	*p*
Age				
versus < 50	≥ 50	1.70	0.75-3.90	0.21
Donor/Recipient Gender				
versus Female	Male	0.81	0.29-2.27	0.70
PS				
versus 0-1	2-4	0.95	0.34-2.68	0.92
Risk				
versus Adverse	Favorable	1.18	0.35-4.04	0.79
	Intermediate	0.69	0.32-1.50	0.35
Conditioning				
versus MAC	RIC	2.08	0.85-5.07	0.11
GVHD prophylaxis				
versus CyA-based	Tac-based	1.48	0.45-4.91	0.52
Source				
versus Bone marrow	Peripheral blood	0.74	0.25-2.15	0.58
	Cord blood	0.74	0.31-1.76	0.50
Remission status				
versus CR	NonCR	10.24	4.18-25.07	<0.001

HR: Hazard Ratio, CI: Confidential Interval, PS: Performance status, MAC: Myeloablative conditioning, RIC: Reduced-intensity conditioning, GVHD: graft-versus-host disease, Tac: Tacrolimus, CyA: Cyclosporine A, CR: complete remission, V/A: Venetoclax plus Azacitidine.

**Table 5.** Multivariate Analysis for incidence of relapse.

		HR	95% CI	*p*
Age				
versus < 50	≥ 50	0.30	0.12-0.78	0.01
Donor/Recipient Gender				
versus Female to Male	Others	0.34	0.13-0.87	0.03
PS				
versus 0-1	2-4	1.22	0.25-5.92	0.80
Risk				
versus Adverse	Favorable	0.23	0.04-1.32	0.10
	Intermediate	0.31	0.11-0.87	0.03
Conditioning				
versus MAC	RIC	3.90	1.53-9.93	0.004
GVHD prophylaxis				
versus CyA-based	Tac-based	2.66	0.93-7.57	0.07
Source				
versus Bone marrow	Peripheral blood	1.61	0.50-5.16	0.43
	Cord blood	0.50	0.20-1.30	0.16
Remission status				
versus CR	NonCR	11.38	4.33-29.91	<0.001

HR: Hazard Ratio, CI: Confidential Interval, PS: Performance status, MAC: Myeloablative conditioning, RIC: Reduced-intensity conditioning, GVHD: graft-versus-host disease, Tac: Tacrolimus, CyA: Cyclosporine A, CR: complete remission.

**Table 6.** Multivariate Analysis for non-relapse mortality.

		HR	95% CI	*p*
Age				
versus < 50	≥ 50	11.48	1.53-85.98	0.02
Donor/Recipient Gender				
versus Female to Male	Others	0.78	0.11-5.26	0.79
PS				
versus 0-1	2-4	2.40	0.62-9.26	0.20
Risk				
versus Adverse	Favorable	4.29	0.77-23.79	0.10
	Intermediate	2.35	0.80-6.93	0.12
Conditioning				
versus MAC	RIC	0.31	0.09-1.10	0.07
GVHD prophylaxis				
versus CyA-based	Tac-based	0.51	0.26-1.00	0.05
Source				
versus Bone marrow	Peripheral blood	0.43	0.04-4.57	0.48
	Cord blood	1.25	0.26-6.08	0.78
Remission status				
versus CR	NonCR	5.24	1.14-24.06	0.03

HR: Hazard Ratio, CI: Confidential Interval, PS: Performance status, MAC: Myeloablative conditioning, RIC: Reduced-intensity conditioning, GVHD: graft-versus-host disease, Tac: Tacrolimus, CyA: Cyclosporine A, CR: complete remission.

## 4. Discussion

The present study is the first multi-center retrospective analysis on thet use of V/A for pre-transplant therapy in Japan. We demonstrated that V/A is a viable option for patients who did not respond to or were unable to tolerate IC. Furthermore, we showed that patients resistant to both IC and V/A had extremely poor transplant outcomes.

The transplant outcomes of patients who achieved CR prior to SCT with IC and V/A did not differ in the present study. V/A is feasible for patients with some comorbidities^22^ and it provides high rate of hematological CR.^6^ Use of V/A for adverse-risk AML prior to transplantation will expand in the future, especially for patients who are refractory or intolerant to IC.

Not only OS and CIR, but also NRM of patients resistant to both IC and V/A were poor in the present study. AML refractory to both IC and V/A may be extremely malignant and difficult to cure through graft-versus-leukemia effect. For this type of AML, development of novel agents or innovative cellular therapies is desirable.

Severe non-hematologic toxicity by V/A should not be underestimated. In the phase 3 trial of V/A, the incidences of severe pneumonia and sepsis were 16% and 6%, respectively.^6^ In the phase 1/2 trial of V/A in Japan, one of six patients developed grade 3 fungal pneumonia, requiring dose interruption of venetoclax and delay of azacitidine.^23^ Moreover, in the retrospective analyses, incidence of adverse cardiac events was comparable in patients receiving venetoclax or anthracyclines.^24,25^ It is possible that these toxicities may affect infection and organ failure after transplant. When V/A is used prior to SCT, where high NRM is anticipated, it may be advisable to perform SCT while paying close attention to prevent infection and organ failure. One important point to be aware of is that most patients who received V/A had undergone multiple cycles of ICs before and after V/A in the present study. These ICs may also have strongly influenced NRM after transplant. In the current study, it was difficult to evaluate the impact of V/A itself on NRM.

In our series, only 6 patients received V/A as induction, and we were unable to identify types of AML in which transplant outcomes were significantly better with pre-transplant V/A than with IC. However, a trend towards better outcomes with V/A was observed in AML with *TP53* mutations, and similar results were reported from the U.S.^26^ Further large-scale studies are warranted to clarify the types of AML patients who should receive V/A prior to SCT. Molecular classification and targeted therapies for AML have been important in recent years.^27^ Quizartinib plus IC for untreated FLT3-positive AML^28^ was approved in 2023, and CPX-351 for adverse-risk AML^29^ was approved in 2024 in Japan. Treatment strategy for AML will become more complex, and its optimization is needed.

Recently, many kinds of molecular targeted drugs have been used for maintenance therapy after SCT.^30^ Maintenance therapy with V/A after SCT is also an important topic. Hypomethylating agents upregulate several antigens on leukemic cells and enhance T cell mediated antitumor activity.^31^ It was reported that prophylactic maintenance with V/A after SCT for high-risk MDS and AML showed a manageable safety profile and encouraging responses.^32^ In the present study, only 7 patients received maintenance, and 2 AML patients with *TP53* received V/A maintenance and survived without relapse. It is awaited to verify efficacy and safety of maintenance with V/A after SCT through randomized trials.

There are several limitations to the present study. First, it is a retrospective analysis on cases who underwent transplant. There were patients who attempted to reach transplantation but were not successful, due to refractoriness to or severe toxicity of chemotherapy; including these patients in a prospective study would help to optimize treatment. Second, the number of participating centers and patients was small, and the observation period was short. Because of the need for quick survey by physicians, the number of participating facilities was limited to 17. We believe that it is important to report research findings of novel drugs in Japan at an early stage and, therefore, decided to publish our findings when the median follow-up period for survivors was approximately one year. Impact of V/A prior to SCT on transplant outcomes will become clearer in the future when the number of patients who receive V/A increases, and the observation period is extended. Third, some of the results in the current study may be affected by bias. The 1-year OS was low in the NonCR groups, and it is presumed that many patients died before reaching the peak period for the development of chronic GVHD, resulting in a low incidence of chronic GVHD. It is possible that physicians selected female donors for male adverse-risk AML patients to induce graft-versus-leukemia effect, and this may explain the association between a high relapse rate and female-to-male SCT observed in the multivariate analysis. Fourth, information that could potentially affect transplant outcomes — such as the percentage of myeloblasts at SCT and dose adjustment for V/A — was not obtained. More detailed examination through prospective research is desired.

In conclusion, use of V/A prior to SCT is feasible and can salvage some patients who were refractory/intolerant to IC, resulting in favourable transplant outcomes. Further study is needed to identify characteristics of AML patients who should receive V/A before SCT.

### Author Contribution

Conceptualization: Hiroyuki Muranushi (Lead). Methodology: Hiroyuki Muranushi (Lead). Formal Analysis: Hiroyuki Muranushi (Lead). Investigation: Hiroyuki Muranushi (Lead). Writing – original draft: Hiroyuki Muranushi (Lead). Resources: Hiroyuki Muranushi (Equal), Ikue Okamura-Shiki (Equal), Takashi Ikeda (Equal), Hiroki Amagase (Equal), Takehiro Okuda (Equal), Daishi Nakagawa (Equal), Takeshi Maeda (Equal), Yasunori Ueda (Equal), Kazunori Imada (Equal), Kazuhiro Yago (Equal), Tomoharu Takeoka (Equal), Mutsumi Okada (Equal), Yasuko Miyahara (Equal), MItsumasa Watanabe (Equal), Nobuyoshi Arima (Equal), Toshiyuki Kitano (Equal), Masakatsu Hishizawa (Equal), Katsuhiro Io (Equal), Satoko Oka (Equal), Kosuke Asagoe (Equal), Takashi Akasaka (Equal), Kouhei Yamashita. Writing – review & editing: Junya Kanda (Equal), Tadakazu Kondo (Equal), Yasuyuki Arai (Equal), Takeshi Maeda (Equal). Supervision: Akifumi Takaori-Kondo (Lead).

### Competition of Interest – COPE

Junya Kanda, Tadakazu Kondo, Yasuyuki Arai, Takeshi Maeda, Kazunori Imada and Katsuhiro Io received honoraria from Nippon Shinyaku and AbbVie. Hiroyuki Muranushi received honoraria from Nippon Shinyaku. Akifumi Takaori-Kondo received honoraria and scholarship grands from Nippon Shinyaku and AbbVie. The other authors declare that they have no conflicts of interests for the present study.

### Ethical Conduct Approval – Helsinki – IACUC

The present study was approved by the Institutional Review Board and Ethics Committee of Kyoto University (R1085).

### Consent to participate / Consent to publish

Written informed consent was obtained from all participating patients.

## Data Availability

The datasets analyzed in the present study are available from Hiroyuki Muranushi on reasonable request.
